# Title, Table of Contents and Acknowledgements

**DOI:** 10.1080/26410397.2020.1888492

**Published:** 2021-03-01


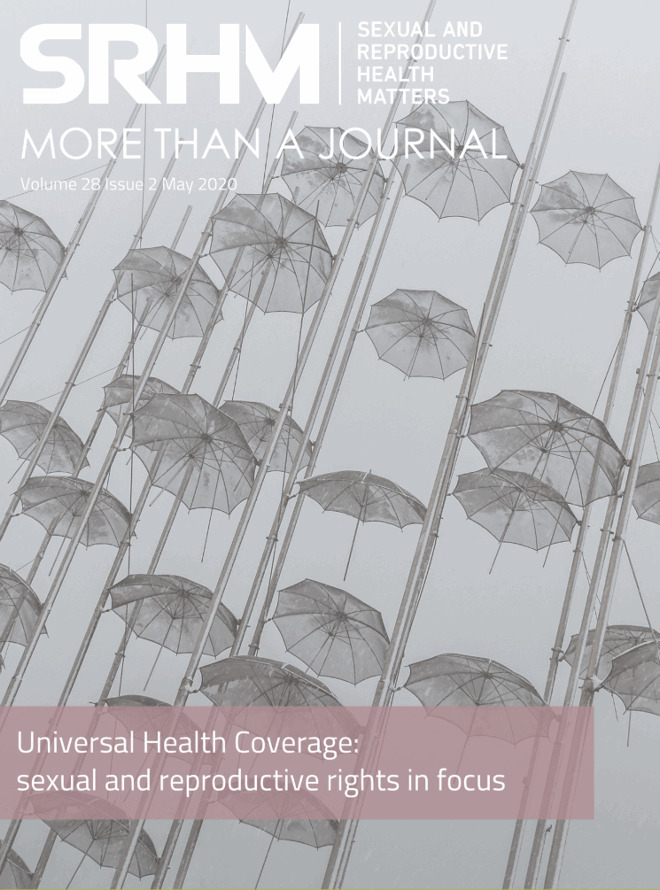


**Editorial**

1  Anna Gruending, Pete Chapman, Veloshnee Govender  “Imagining the world anew”: a transformative, rights-based agenda for UHC and SRHR in 2021 and beyond

**Commentaries**

7  Nino Berdzuli, Mikael Ostergren, Ketevan Chkhatarashvili, Martin W Weber, Susanne Carai  Sexual and reproductive health and rights: still ground zero for UHC in the WHO European Region

11  Helen Clark, Anna Gruending  Invest in health and uphold rights to “build back better” after COVID-19

16  Katharine Footman, Banchiamlack Dessalegn, George Hayes, Kathryn Church  Can universal health coverage eliminate unsafe abortion?

22  Lynda Gilby, Meri Koivusalo  Universal health coverage: another political space in which to expand the elimination of sexual and reproductive health and reproductive rights

26  Felicita Hikuam, Tamaryn L. Crankshaw, Jesper Sundewall  Engaging communities: the key to leaving no one behind in the era of UHC

30  Tanvi Monga, Madhabi Bajracharya, Hina Aziz, Lhamo Yangchen Sherpa, Irum Shaikh, Ghulam Shabbir, Popular Gentle, Ghazaleh Samandari  Increasing safe abortion access through universal health care: promising signs from Nepal and Pakistan

34  Warisa Panichkriangkrai, Chompoonut Topothai, Nithiwat Saengruang, Jadej Thammatach-aree, Viroj Tangcharoensathien   Universal access to sexual and reproductive health services in Thailand: achievements and challenges

40  Yogan Pillay, Gerald Manthalu, Hakiba Solange, Velephi Okello, Mikaela Hildebrand, Jesper Sundewall, Eoghan Brady  Health benefits packages: moving from aspiration to action for improved access to quality SRHR through UHC reforms

44  Chelsea L. Ricker, Rebekah Ashmore  The importance of power and agency in a universal health coverage agenda for adolescent girls

48  Lisa Seidelmann, Myria Koutsoumpa, Frederik Federspiel, Mit Philips  The Global Financing Facility at five: time for a change?

56  Cynthia Khamala Wangamati  Comprehensive sexuality education in sub-Saharan Africa: adaptation and implementation challenges in universal access for children and adolescents

**Perspective**

62  Faysal El Kak  The integration of sexual and reproductive health and rights into universal health coverage: a FIGO perspective

**Reviews**

65  Gabrielle Appleford, Saumya RamaRao, Ben Bellows  The inclusion of sexual and reproductive health services within universal health care through intentional design

76  Tamar Kabakian—Khasholian, Harumi Quezada—Yamamoto, Ahmed Ali, Shible Sahbani, Mohamed Afifi, Salman Rawaf, Maha El Rabbat  Integration of sexual and reproductive health services in the provision of primary health care in the Arab States: status and a way forward

89  Lisa Owino, Annette Wangong'u, Nerima Were, Allan Maleche  The missing link in Kenya's universal health coverage experiment: a preventive and promotive approach to SRHR

106  T. K. Sundari Ravindran, Veloshnee Govender  Sexual and reproductive health services in universal health coverage: a review of recent evidence from low- and middle-income countries

**Research articles**

128  James Akazili, Edmund Wedam Kanmiki, Dominic Anaseba, Veloshnee Govender, Georges Danhoundo, Augustina Koduah  Challenges and facilitators to the provision of sexual, reproductive health and rights services in Ghana

143  Bouchra Assarag, El Omrani Sanae, Bezad Rachid  Priorities for sexual and reproductive health in Morocco as part of universal health coverage: maternal health as a national priority

159  Sharyn Graham Davies, Najmah  Im/moral health care: HIV and universal health coverage in Indonesia

176  Veena Iyer, Dileep Mavalankar, Rachel Tolhurst, Ayesha De Costa  Perceptions of quality of care during birth at private Chiranjeevi facilities in Gujarat: lessons for Universal Health Coverage

193  Clara Juárez-Ramírez, Gustavo Nigenda, Alma L. Sauceda-Valenzuela, Aremis Villalobos  Lags in the provision of obstetric services to indigenous women and their implications for universal access to health care in Mexico

211  Godfrey Kangaude, Ernestina Coast, Tamara Fetters  Adolescent sexual and reproductive health and universal health coverage: a comparative policy and legal analysis of Ethiopia, Malawi and Zambia

226  Shiang Cheng Lim, Yee Chern Yap, Sima Barmania, Veloshnee Govender, Georges Danhoundo, Michelle Remme  Priority-setting to integrate sexual and reproductive health into universal health coverage: the case of Malaysia

250  Naomi Lince-Deroche, Elizabeth A Sully, Lauren Firestein, Taylor Riley  Budgeting for comprehensive sexual and reproductive health and rights under universal health coverage

269  Muriel Mac-Seing, Kate Zinszer, Bryan Eryong, Emma Ajok, Olivier Ferlatte, Christina Zarowsky  The intersectional jeopardy of disability, gender and sexual and reproductive health: experiences and recommendations of women and men with disabilities in Northern Uganda

284  Najmah, Sari Andajani, Sharyn Graham Davies  Perceptions of and barriers to HIV testing of women in Indonesia

298  Manjulaa Narasimhan, Carmen H. Logie, Alice Gauntley, Rodolfo Gomez Ponce de Leon, Karima Gholbzouri, Nandi Siegfried, Heather Abela, Leopold Ouedraogo  Self-care interventions for sexual and reproductive health and rights for advancing universal health coverage

315  Bang Nguyen Pham, Maxine Whittaker, Anthony D. Okely, William Pomat  Measuring unmet need for contraception among women in rural areas of Papua New Guinea

332  Lucas Godoy Garraza, Federico Tobar, Iván Rodríguez Bernate  Out-of-pocket spending for contraceptives in Latin America

342  Wan-Ju Wu, Aparna Tiwari, Nandini Choudhury, Indira Basnett, Rita Bhatt, David Citrin, Scott Halliday, Lal Kunwar, Duncan Maru, Isha Nirola, Sachit Pandey, Hari Jung Rayamazi, Sabitri Sapkota, Sita Saud, Aradhana Thapa, Alisa Goldberg, Sheela Maru  Community-based postpartum contraceptive counselling in rural Nepal: a mixed-methods evaluation

**Editor-in-Chief:** Julia Hussein

**Chief Executive**: Eszter Kismödi

**Managing Editor**: Pete Chapman

**Monitoring Editor**: Pathika Martin

**South Asia Hub Manager**: Sanjeeta Gawri

**Communications Manager**: Jessica MacKinnon

**Communications Officer**: Alexane Bremshey

**Operations Manager**: Edna Epelu/Amy Guthrie

**Associate Editors:** Laura Ferguson, Nambusi Kyegombe, Atsumi Hirose, Emma Pitchforth, Mindy Jane Roseman, Nina Sun, Joyce Wamoyi

**Translation:**

Françoise de Luca-Lacoste translated abstracts from English to French and Lisette Silva translated abstracts from English to Spanish.

**Cover photo:**

Snowy day on New Beach, Thessaloniki, Greece. Photographer: Chris Karidis

**Funding:** This themed issue has been funded by The Partnership for Maternal, Newborn & Child Health (PMNCH) and the UNDP-UNFPA-UNICEF-WHO-World Bank Special Programme of Research, Development and Research Training in Human Reproduction (HRP), a cosponsored programme executed by the World Health Organization (WHO). Authors are responsible for the content of their articles which do not necessarily reflect positions or policies of the funders


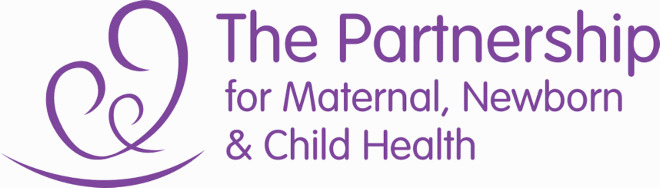


**Peer reviewers:**

Barbara Baird, Ben Bellows, Helen Benedict Lasimbang, Lethia Bernard, Ann Biddlecom, Adriana Biney, Kirsten I Black, Victoria Boydell, Claire Brolan, Thanh Cong Bui, Irene Capelli, Roberto Castro, Arachu Castro, Nirali Chakraborty, Kathya Lorena Cordova-Pozo, Jane Cottingham, Marilen Danguilan, Blair G Darney, Sharyn Davies, Billie de Haas, Sapna Desai, Nicola Desmond, Anubal Faúndes, Abbey J Hardy-Fairbanks, Katharine Footman, Alexandra Gartrell, Ursula Giedion, Jessica Gipson, Veloshnee Govender, Jamison Green, Sofia Gruskin, Pande Putu Januraga, Austin Johnson, Gary Jones, Rachel Joness, Melissa Kang, Lucy Kanya, Louise A Keogh, Evert Ketting, Michael Kunnujio, Mona Loutfy, Priscilla Magrath, Anita Makins, Laura Miranda, Glen Liddell Mola, Karen Marie Moland, Emmanuel Morhe, Wisdom Kwadwo Mprah, Jenny Munro, Manjulaa Narasimhan, Ann Nolan, Emilomo Ogbe, Kristine Husey Onarheim, Rose Ndakala Oronje, Julie Pannetier, Katrina Perehudoff, Anne Pfitzer, James Phillips, Billie Powell, Martha Rac, Sundari Ravindran, Laura Reichenbach, Michelle Remme, Mario Reyes Festin, Felix Rigoli, Rachel Robinson, James Rosen, Sam Rowlands, Ana Lorena Ruano, Nicole Salisbury, Frederic Seghers, Gamal Serour, Bonnie K Shepard, Ronli Sifris, Ashish Srivastava, Jesper Sundewall, Joar Svanemyr, Sivananthi Thanenthiran, Jurrien Toonen, Ashley Vandermorris, Anna Whelan, Lisa Wynn

**Copyright ©2021 Sexual and Reproductive Health Matters:** This is an Open Access journal distributed under the terms of the Creative Commons Attribution License (http://creativecommons.org/licenses/by/4.0/), which allows for sharing and adapting the work for any purpose, even commercially, provided appropriate credit is given with a link to the originally published item, a reference to the author(s) and links to their homepages, reference to the license under which the article is published and a link to this, as well as an indication of any changes that have been made to the original. ISSN (Online) 2641-0397

